# Efficacy of Cell-Based Therapies for Traumatic Brain Injuries

**DOI:** 10.3390/brainsci9100270

**Published:** 2019-10-10

**Authors:** Matthew R. Chrostek, Emily G. Fellows, Winston L. Guo, William J. Swanson, Andrew T. Crane, Maxim C. Cheeran, Walter C. Low, Andrew W. Grande

**Affiliations:** 1 Department of Neurosurgery, University of Minnesota Medical School, Minneapolis, MN 55455, USA; chros005@umn.edu (M.R.C.); fello064@umn.edu (E.G.F.); guoxx838@umn.edu (W.L.G.); swan2166@umn.edu (W.J.S.); atcrance@umn.edu (A.T.C.); lowwalt@umn.edu (W.C.L.); 2 Department of Veterinary Population Medicine, University of Minnesota College of Veterinary Medicine, St. Paul, MN 55108, USA; cheeran@umn.edu; 3 Stem Cell Institute, University of Minnesota Medical School, Minneapolis, MN 55455, USA

**Keywords:** traumatic brain injury, stem cells, inflammation, neuroregeneration, mesenchymal stem cells, neural stem cells

## Abstract

Traumatic brain injuries (TBIs) are a leading cause of death and disability. Additionally, growing evidence suggests a link between TBI-induced neuroinflammation and neurodegenerative disorders. Treatments for TBI patients are limited, largely focused on rehabilitation therapy, and ultimately, fail to provide long-term neuroprotective or neurorestorative benefits. Because of the prevalence of TBI and lack of viable treatments, new therapies are needed which can promote neurological recovery. Cell-based treatments are a promising avenue because of their potential to provide multiple therapeutic benefits. Cell-based therapies can promote neuroprotection via modulation of inflammation and promote neurorestoration via induction of angiogenesis and neurogenesis. Neural stem/progenitor cell transplantations have been investigated in preclinical TBI models for their ability to directly contribute to neuroregeneration, form neural-like cells, and improve recovery. Mesenchymal stem cells (MSCs) have been investigated in clinical trials through multiple different routes of administration. Intravenous administration of MSCs appears most promising, demonstrating a robust safety profile, correlation with neurological improvements, and reductions in systemic inflammation following TBI. While still preliminary, evidence suggests cell-based therapies may become a viable treatment for TBI based on their ability to promote neuroregeneration and reduce inflammation.

## 1. Introduction

Each year in the United States, traumatic brain injuries (TBIs) lead to 53,000 deaths [1]. Additionally, TBIs are the primary cause of disability for 5.3 million people within the United States, making TBI the leading cause of injury-related death and disability [1]. TBI-related healthcare expenditures cost the United States an estimated 60 billion dollars each year, largely attributed to the costs of short- and long-term disability [2]. TBI-related disability is linked to the severity of the initial injury and the following neuroinflammatory response which may persist long after the initial injury [3]. TBI and the ensuing neuroinflammation, in addition to causing motor and cognitive deficits, have been linked to increased risk of neurodegenerative disorders including Alzheimer’s disease, Parkinson’s disease, amyotrophic lateral sclerosis, and chronic traumatic encephalopathy [4]. Treatments to improve recovery after TBI are limited largely to rehabilitation, which may address some deficits [5] but ultimately fail to provide neuroprotective or neurorestorative benefits. To ameliorate the deficits caused by TBI, therapies must address the initial injury to the brain and the ensuing inflammatory response.

TBI damage and break down of the blood brain barrier (BBB) enables infiltration of peripheral immune cells [3]. The initial injury induces an inflammatory response in order to fight infection and promote wound healing [6]. This response includes complement activation which accompanies recruitment of inflammatory immune cells across the BBB [6,7]. Responding immune cells secrete prostaglandins, free radicals, and proinflammatory mediators, which increase expression of chemokines and cell adhesion molecules, leading to infiltration of activated immune cells into the brain parenchyma [7,8]. Activated microglia manifest as different functional phenotypes [9], which on one hand can mount a protective response following TBI that limits the spread of damage and promotes recovery, but on the other hand, may become pro-inflammatory by releasing neurotoxic molecules and proinflammatory cytokines resulting in secondary damage [10]. In addition, up-regulation of pro-inflammatory cytokines by microglia increases BBB permeability and expression of cell adhesion molecules and chemokines, further increasing immune cells infiltration and augmenting the damaging consequences of the inflammatory response [11]. Microglia play a critical role in driving neuroinflammation and may serve as a target both for monitoring and modulating the neuroinflammatory response to TBI [12,13]. This inflammatory cascade, initiated by TBI, may persist and become amplified in the brain, predisposing TBI patients to neurological decline and neurological disorders [14,15].

Clinical trials have yet to yield neuroprotective agents suitable for treating neuroinflammation in TBI patients, likely because of the limited scope of drug-based approaches attempting to reduce single components of the complex inflammatory cascade [16]. Even though persistent neuroinflammation is associated with worse outcomes, the initial inflammation is necessary for healing, and so treatments must aim to reduce the negative effects of inflammation while enhancing or at least not impeding upon the healing effects [17]. Additionally, altering a single inflammatory component may not be enough because of the complex and amplificatory nature of the neuroinflammatory response [16].

Cell-based therapies offer an alternative, with the potential to not only modulate systemic inflammation but also provide multiple neurorestorative benefits by simultaneous promotion of neurogenesis, angiogenesis, and neuroprotection at the site of injury [18]. Two prominent cell types, neural stem/progenitor cells (NSPCs) and mesenchymal stem cells (MSCs), have demonstrated the ability to improve neurological outcomes and recovery in preclinical trials investigating cerebral injury models. NSPCs are thought to mediate their effects through cell replacement via differentiation into neurons in the injured region, as well as through secretion of glial cell-derived neurotrophic factor and other neuroprotective factors [19]. MSCs have been shown to improve neurological recovery in multiple central nervous system (CNS) injury models as well, including TBI [16]. The therapeutic effects of MSCs are linked to their ability to modulate the inflammatory response and secrete neurotrophic factors which promote the protection and development of neurons [18]. Here, we explore both preclinical and clinical work on NSPC- and MSC-based therapies for TBI.

## 2. Neural Progenitor Cell Therapy for TBI

Studies of embryonic stem cells demonstrated that fetal tissue could be used to derive NSPCs, which had the ability to differentiate into neurons and innervate recipient brains, suggesting fetal-derived NSPCs could be used to replace neurons lost to TBI and improve recovery [20]. In TBI rat models, intracranial rat NSPC transplants survived and differentiated into functioning neurons, which correlated with reductions in glial scar size and improved neurological motor functions [21]. The therapeutic effects of rat NSPCs and their contribution to the formation of additional neurons following transplantation could additionally be enhanced through infusion of nerve growth factor [22] or through seeding of NSPCs in a transplanted scaffold [23]. Studies in mice also demonstrated that mouse NSPC transplants could survive upwards of 14 months and contributed to long-term improvements in memory [24].

Preclinical work using human NSPCs in TBI rat models showed that human NSPCs were capable of surviving engraftment and differentiating into neurons which, in turn, correlated with improvements in neurological recovery. Additionally, this work demonstrated that long-term cultured and cryopreserved human NSPCs were suitable for transplantation and produced similar improvements in neurological recovery [25]. While human NSPC transplants have shown the ability to alleviate motor deficits in TBI models, long-term cognitive and memory improvements are lacking [26], suggesting room for improvement. Although NSPC therapies have not been examined in clinical trials as a treatment for TBI, the series of PISCES trials have explored NSPC therapy for treatment of ischemic stroke, which was proven safe [27]. Based on promising results the PISCES III trial (NCT03629275) is now underway to examine the efficacy of NSPC therapy for stroke patients. Because of the parallels between brain damage caused by stroke and TBI, if NSPC therapy proves effective in stroke patients, it may be readily translatable to patients suffering from deficits caused by TBI, as evidenced in stroke and TBI animal models [28].

## 3. Mesenchymal Stem Cell Therapies for TBI

MSCs are a promising cellular therapy due to their properties of multipotency and self-renewal, as well as ease of isolation and propagation [29]. MSCs can be found in most tissues and are typically harvested from adipose tissue, peripheral blood, the umbilical cord, or bone marrow, with the latter predominating investigation for clinical use [30]. Bone marrow mesenchymal stem cells (BMSCs) are a heterogenous population of cells which provide support for hematopoietic cells and contain stem cells capable of differentiating into bone, cartilage, adipocytes, and hematopoietic supporting cells [29]. Based on their differentiation potential and accessibility, BMSCs were first explored in transplant studies to treat ischemic stroke rat models under the hypothesis that transplanted BMSCs would differentiate into neurons and reconstitute damaged regions of the brain. Intracerebral transplantation of BMSCs into the infarct region of ischemic stroke rat models led to improvements in functional recovery. However, only a fraction of transplanted cells survived, of which only a small number displayed neuronal-like markers, indicating functional recovery was not linked to the generation of new replacement neurons from the BMSCs [31,32]. BMSC intracranial transplants showed a similar ability to improve functional recovery in TBI rat models, but again yielded few surviving neuronal-like cells, implicating another mechanism of action [33].

Because the effects of BMSCs seemed independent of their ability to form new neurons in the damaged region of the brain, researchers began exploring the trophic and neurotrophic effects of BMSC. In treating TBI, re-establishing blood flow to the injured area is critical for cellular growth and recovery. Endothelial progenitor cells (EPCs) promote angiogenesis through tissue repair and can be mobilized under various stimulating factors, including vascular endothelial growth factor (VEGF) [34]. However, VEGF and other vascular growth factor levels are reduced following TBI [35]. A multitude of studies have demonstrated that BMSCs have an angiogenic effect in promoting neurologic recovery in TBI animal models. Hu et al. found that BMSC transplantation increased the number of EPCs in the peripheral blood of rats with TBI 24 hours after injury, which correlated with improved modified neurological severity scores (mNSS) compared to controls [34]. This effect was also investigated by Guo et al., who demonstrated that BMSCs administered intravenously post-TBI upregulated VEGF and angiogenin-1 levels in a rat controlled cortical impact (CCI) TBI model. These changes were correlated with the formation of microvessels [35]. These results emphasize the importance of angiogenesis in improving neurologic outcome after TBI and the role BMSCs play in facilitating this restorative process.

In addition to promoting angiogenesis, BMSCs have been shown to ameliorate neuronal dysfunction induced by TBI through the production of neurotrophic factors, which promote survival and normal growth of surviving neurons. Although neuronal cell death is a major contributor to poor neurologic outcome post-TBI, surviving neurons have been shown to exhibit changes in dendritic morphology, decreased dendritic branches, decreased density of dendritic spines, and decreased density of synapses in a mouse CCI TBI model [36]. Addressing this cellular dysfunction in surviving neurons can help improve neurologic recovery. A study by Feng et al. found that administration of BMSCs via tail vein injection in a weight-drop rat TBI model promoted neuromotor recovery via upregulation of neurotrophic factors (VEGF and BDNF) and synaptic proteins (synaptophysin) in the brain. Thus, BMSCs not only helped restore the synaptic function of surviving neurons but also promoted neuroregeneration [37].

In regards to long-term therapeutic efficacy of BMSCs, Mahmood et al. found that rat BMSCs injected intravenously one week post-TBI in a rat model survived in the recipient animal three months post-treatment. In addition, functional improvements and growth factor production continued to be observed at three months post-BMSC administration [38]. Because improvements persisted despite declining numbers of BMSC, this study highlights that functional recovery may not be directly correlated with BMSC graft survival.

Researchers have also investigated the migration-capabilities of BMSCs in order to understand where the cells home to and whether BMSCs can be therapeutically applied through techniques less invasive than intracranial transplants. Early work showed that following intra-arterial infusion in rats with TBI BMSCs migrated to the brain, but no functional recovery was observed. The lack of therapeutic effect was attributed to the route of administration, where ligation of the internal carotid artery may have induced hypoperfusion, unintentionally exacerbating the initial injury [39,40]. Exploring an alternative route, using intravenous delivery in a TBI rat model, BMSCs also migrated to the parenchyma of the injured brain and, in this study, were associated with functional recovery including improved mNSS and rotarod performance [41]. Work exploring the mechanism of BMSC migration revealed that inflammatory cytokines and chemokines generated by cerebral injury recruited BMSCs from the periphery. Human BMSCs have also been tested in TBI rat models where they were found to demonstrate similar migration patterns and improve functional recovery [40]. Based on the success of rodent and human BMSCs in improving neurological recovery in TBI animal models, efforts are now underway to evaluate BMSCs in the clinic.

In addition to treatment using BMSCs, MSCs have been harvested from other tissue sources in efforts to explore whether the tissue source of MSCs impacts their therapeutic effects. MSCs have been derived from human peripheral blood that were shown to produce neurotrophic factors and when administered to a TBI rat model, reduced apoptosis due to injury [42]. Compared to autologous bone-marrow-derived treatments, MSC derived from peripheral blood offers a less invasive autologous treatment. However, peripheral-blood-derived MSCs have yet to demonstrate improvements to cognitive function in TBI models [42], a significant hurdle which must be cleared before replacing BMSC as a viable therapeutic alternative.

Of growing interest are MSCs derived from human umbilical cord blood (hUCB). hUCB, when administered intravenously, was able to reduce motor and neurological deficits in TBI rat models [43]. Further examination of hUCB revealed populations of MSCs with anti-inflammatory properties [44]. MSCs derived from hUCB have since been tested as treatments for stroke where they demonstrated the ability to reduce injury infarct volume and improve neurological recovery, which correlated with reductions in both systematic and neuroinflammation [45,46]. The ability for human hUCB MSCs to modulate inflammation and improve recovery in cerebral injury models suggests it may be a promising avenue to explore for the treatment of TBI patients.

### 3.1. Autologous MSC Therapies in Clinical Research

Based on the results of preclinical work, autologous BMSC therapies for cerebral injuries have slowly made their way into clinical trials, first in stroke, and now TBI. In both adults and children with TBI, intravenous administration of autologous BMSCs was proven to be safe [47,48,49]. There were no adverse events related to collection or administration of BMSCs, however, dose-related pulmonary toxicity was observed in adults when administering more than 9 × 10^6^ cells/kg [49], suggesting a limit which must be accounted for in future trials assessing efficacy. Additional routes for autologous BMSC treatments have been explored, including lumbar puncture and intracerebral transplantation. Trials investigating BMSC administration via lumbar puncture and intracerebral transplantation showed no serious adverse events during collection and administration of BMSCs or during follow-up, providing support for autologous BMSCs as a safe treatment for TBI [47,50].

While autologous BMSC therapy for TBI has proven safe, the small number of trials limits conclusions about efficacy. Combinatorial BMSC therapy for seven patients, using intracerebral transplantations and intravenous injections, showed improvements in recovery via improved Barthel index scores, a measure for daily living and functional independence, over the course of six months [47]. Intravenous injection of BMSCs alone has also been correlated with improved recovery, as well as a reduction in systemic inflammatory markers in the blood, including TNF-α, IL-1β, IL-10, and IFN-γ [48,49]. No significant improvement in long-term recovery was observed after autologous BMSC therapy via lumbar puncture in TBI patients [50]. Based on the limited clinical data, intravenous BMSC therapy appears to be the most promising route for treating TBI because of its safety, relatively low level of invasiveness, and correlation with improved recovery and reduced inflammatory biomarkers [49].

### 3.2. Non-Autologous MSC Therapies in Clinical Research 

While MSC-based therapies have typically utilized MSCs isolated from bone marrow, researchers have also investigated using MSCs derived from other sources, including hUCB. A recent clinical trial evaluated whether allogeneic hUCB MSCs could be used to treat patients with sequelae from TBI. Forty patients were randomly assigned to receive hUCB MSCs via lumbar punctures or no medical treatment (control) and were followed for six months. Compared to controls, the hUCB MSCs treatment group showed significant improvements in Fugl–Meyer Assessment scores, which assess motor function and balance, as well as significant improvements in Functional Independence Measures, which assess self-care and communication abilities. No serious adverse events were noted. This study demonstrated allogeneic hUCB MSC administration via lumbar injection was safe and may be able to alleviate chronic motor deficits in TBI patients [51]. While trials have been limited, an allogeneic MSC-based therapy would provide an “off the shelf” treatment compared to autologous MSC therapies, and assuming similar safety and efficacy profiles, provide a more accessible treatment for patients.

The application of hUCB-based treatments has also been applied in the context of stroke patients, whose neurological damage and deficits often mirror those of TBI. Recently, a phase 1 study treated adult stroke patients with non-HLA-matched hUCB. Patients receiving hUCB all showed improved neurological function and the study yielded no serious treatment-related adverse events suggesting hUCB treatment was safe and potentially efficacious [52]. Based on these results, hUCB-based therapies may provide readily available treatments for neurological injuries providing support for continued investigation into the use of hUCB for treating TBIs.

## 4. Immune Cell Therapies for TBI

While many cell-based approaches have harnessed stem cells for their immunomodulatory properties, researchers have also investigated the use of immune cell transfers as a treatment for modulating neuroinflammation [53]. Approaches investigating the direct transfer of immune cells have homed in on the role and use of regulatory T cells and B cells. In a mouse TBI model, depletion of regulatory T cells leads to an increase in T cell infiltration of the parenchyma following injury, which coincides with increased interferon-γ expression and reactive gliosis, as well as worse motor deficits [54]. Therapies which transfer or enhance endogenous regulatory T cells may be a viable treatment option for reducing T-cell-associated neuroinflammation following TBI. 

Recently the role of B cells in wound healing and dampening inflammation has also been brought to light in the context of TBI. Based off observations that B cells secreted factors which could modulate macrophage and microglia behavior [55], as well as promote the growth of nerves at scar tissue [56], researchers investigated whether B cells could alleviate inflammation and promote healing following TBI. Intracranial injection of B cells immediately prior to TBI in mice led to improved cognitive performance and reduced lesion volume compared to saline-treated controls. Additionally, administered cells persisted for approximately two weeks, indicating transferred cells fail to proliferate and are unlikely to contribute long-term effects (negative or positive) [57]. While still in the preclinical stages, cell-based approaches using immune cells may offer a more direct way of modulating the inflammatory response in TBI patients in order to reduce neuroinflammation and improve outcomes.

## 5. Improving Cell-Based Therapies for TBI

NSPC- and MSC-based therapies have proven beneficial in improving functional outcome in preclinical TBI models and safe in early clinical trials. However, neuroinflammation remains a significant, unaddressed contributor to TBI-induced functional and cognitive decline. Current research efforts have largely evaluated the angiogenic, neuroregenerative, and neuroprotective properties of cell-based therapies, overlooking immunomodulatory effects. Growing evidence, however, suggests that modulation of the immune system may be a key mechanism through which cell-based therapies improve neurological function and recovery following TBI. Work by Cox et al. has now shown increased levels of inflammatory cytokines following TBI in humans, including TNF-α, IL-1β, IL-10, and IFN-γ, which are reduced after treatment with BMSCs [49]. Additionally, work in TBI animal models has highlighted the effectiveness of improving cell-based therapies for TBI by enhancing their immunomodulatory effects.

Efforts to enhance cell-based therapies have investigated strategies which augment the transplanted cells by providing additional anti-inflammatory molecules or which stimulate the transplanted cells to become more active or mobile. Inhibitors of apoptosis and inflammation have been shown to enhance neurological recovery when combined with cell-based therapies. Calpain, one of the first pro-inflammatory cytokines upregulated following neurotrauma, mediates necrotic and apoptotic cell death, contributing to axonal degeneration and oligodendrocyte, as well as motor neuron death. MDL28170, a calpain inhibitor, used as therapy for TBI (in a weight drop rat TBI model), in combination with BMSCs, reduced neuroinflammation and grafted cell apoptosis, improved graft survival, and ultimately improved functional recovery compared to control animals [58]. 

Because hUCB MSCs have shown strong immunomodulatory properties, researchers have also investigated ways to increase their activity and migration. Granulocyte-colony stimulating factor (GCS-F) activates hematopoietic cells [59] and the use of G-CSF-activated hUCB cells to treat a TBI rat model led to a greater reduction in neuroinflammation and improvement in motor recovery when compared to TBI rats treated with hUCBs alone [60].

Rather than administer cells and immunomodulatory molecules as a cocktail, researchers have also investigated whether cells could be genetically engineered to enhance their immunomodulatory and neuroprotective properties. Viral transfection of MSCs has been preferentially used for research purposes because of the ability for viruses to deliver long-term stable transgene expression with high transduction efficiency [61]. In a CCI rat TBI model, lentiviral transduction to induce IL-10 expression in the administered MSCs led to reduced inflammation and improved functional recovery [62]. A study in a mouse ischemic injury model assessing the intranasal application of genetically modified MSCs highlighted the complexities of overexpressing factors normally associated with growth and development. MSCs were transduced with adenoviruses containing *BDNF*, *EGFL7*, *PSP*, or *SHH*. While MSC-BDNF improved motor function, decreased lesion volumes, and induced cell proliferation when compared to controls (MSC-empty vectors), the other modified MSC treatments either failed to provide improvements or even contributed to a decline in motor function. While promising, the complex interplay between the inflammatory cascade and introduction of genetically engineered MSCs secreting increased levels of immunomodulatory, growth, and other trophic factors requires further work to determine viable gene targets or combinations of genes for modification [63]. 

Augmentation of cell-based therapies, specifically the enhancement of their anti-inflammatory properties, has proven effective in treating preclinical TBI models, supporting the need for further work on cell types which demonstrate robust immunomodulatory capabilities. Genetically modified MSCs are growing in popularity and have been proven to be beneficial in animal models, warranting further investigation.

## 6. Conclusions

Over the past decade, a series of clinical trials have begun to investigate the safety and efficacy of cell-based therapies in treating brain damage caused by TBI. These treatments appear to be safe and have demonstrated the ability to improve neurological and motor functions in human TBI patients. However, the mechanisms by which these improvements are mediated remain unknown. Evidence from TBI animal models suggests that reductions in inflammation may lead to recovery, but supporting evidence in human patients is limited. Additional therapeutic mechanisms could include the promotion of angiogenesis and neurogenesis through the secretion of neurotrophic and other factors, or for intracerebral transplantation, differentiation of transplanted cells into new neurons. Although the therapeutic mechanisms remain unclear, these results are promising and suggest that current trials may yield considerable clinical improvements for TBI patients. Moreover, clinical trials using stem cell therapies for other cerebral injuries, such as stroke, are ongoing as well. If successful, these therapies may be translatable to TBI patients because of the parallels in mechanisms of damage and recovery between cerebral injuries like TBI and stroke. 

Future studies are needed to elucidate the mechanisms by which stem cell therapies promote recovery following TBI, as well as evaluate the effectiveness of these therapies. In addition, it will be important to test the effects of immune system modifiers administered with stem cells, as this combined approach could further reduce neuroinflammation and aid recovery. Ultimately, the goal of these studies should be to determine whether existing therapies are capable of protecting the brain and restoring function following TBI.

## References

[1] Centers for Disease Control and Prevention (2015). Centers for Disease Control and Prevention Report to Congress on Traumatic Brain Injury in the United States: Epidemiology and Rehabilitation.

[2] Finkelstein E., Corso P.S., Miller T.R. (2006). The Incidence and Economic Burden of Injuries in the United States.

[3] Hay J.R., Johnson V.E., Young A.M.H., Smith D.H., Stewart W. (2015). Blood-Brain Barrier Disruption Is an Early Event That May Persist for Many Years After Traumatic Brain Injury in Humans. J. Neuropathol. Exp. Neurol..

[4] Gardner R.C., Yaffe K. (2015). Epidemiology of mild traumatic brain injury and neurodegenerative disease. Mol. Cell. Neurosci..

[5] Dang B., Chen W., He W., Chen G. (2017). Rehabilitation Treatment and Progress of Traumatic Brain Injury Dysfunction. Neural Plast..

[6] Schmidt O.I., Heyde C.E., Ertel W., Stahel P.F. (2005). Closed head injury—An inflammatory disease?. Brain Res. Rev..

[7] Fluiter K., Opperhuizen A.L., Morgan B.P., Baas F., Ramaglia V. (2014). Inhibition of the Membrane Attack Complex of the Complement System Reduces Secondary Neuroaxonal Loss and Promotes Neurologic Recovery after Traumatic Brain Injury in Mice. J. Immunol..

[8] Lozano D., Gonzales-Portillo G.S., Acosta S., de la Pena I., Tajiri N., Kaneko Y., Borlongan C.V. (2015). Neuroinflammatory responses to traumatic brain injury: Etiology, clinical consequences, and therapeutic opportunities. Neuropsychiatr. Dis. Treat..

[9] Jassam Y.N., Izzy S., Whalen M., McGavern D.B., El Khoury J. (2017). Neuroimmunology of Traumatic Brain Injury: Time for a Paradigm Shift. Neuron.

[10] Hernandez-Ontiveros D.G., Tajiri N., Acosta S., Giunta B., Tan J., Borlongan C.V. (2013). Microglia activation as a biomarker for traumatic brain injury. Front. Neurol..

[11] Chodobski A., Zink B.J., Szmydynger-Chodobska J. (2011). Blood-brain barrier pathophysiology in traumatic brain injury. Transl. Stroke Res..

[12] Cox C.S., Juranek J., Bedi S. (2019). Clinical trials in traumatic brain injury: Cellular therapy and outcome measures. Transfusion.

[13] Savitz S.I., Cox C.S. (2016). Concise Review: Cell Therapies for Stroke and Traumatic Brain Injury: Targeting Microglia. Stem Cells.

[14] Ramlackhansingh A.F., Brooks D.J., Greenwood R.J., Bose S.K., Turkheimer F.E., Kinnunen K.M., Gentleman S., Heckemann R.A., Gunanayagam K., Gelosa G. (2011). Inflammation after trauma: Microglial activation and traumatic brain injury. Ann. Neurol..

[15] Glushakova O.Y., Johnson D., Hayes R.L. (2014). Delayed Increases in Microvascular Pathology after Experimental Traumatic Brain Injury Are Associated with Prolonged Inflammation, Blood–Brain Barrier Disruption, and Progressive White Matter Damage. J. Neurotrauma.

[16] Xiong Y., Mahmood A., Chopp M. (2018). Current understanding of neuroinflammation after traumatic brain injury and cell-based therapeutic opportunities. Chin. J. Traumatol..

[17] Ziebell J.M., Morganti-Kossmann M.C. (2010). Involvement of pro- and anti-inflammatory cytokines and chemokines in the pathophysiology of traumatic brain injury. Neurother. J. Am. Soc. Exp. Neurother..

[18] Walker P.A., Harting M.T., Shah S.K., Day M.C., El Khoury R., Savitz S.I., Baumgartner J., Cox C.S. (2010). Progenitor cell therapy for the treatment of central nervous system injury: A review of the state of current clinical trials. Stem Cells Int..

[19] Yan Z.J., Zhang P., Hu Y.Q., Zhang H.T., Hong S.Q., Zhou H.L., Zhang M.Y., Xu R.X. (2013). Neural Stem-Like Cells Derived from Human Amnion Tissue are Effective in Treating Traumatic Brain Injury in Rat. Neurochem. Res..

[20] Weston N.M., Sun D. (2018). The Potential of Stem Cells in Treatment of Traumatic Brain Injury. Curr. Neurol. Neurosci. Rep..

[21] Riess P., Zhang C., Saatman K.E., Laurer H.L., Longhi L.G., Raghupathi R., Lenzlinger P.M., Lifshitz J., Boockvar J., Neugebauer E. (2002). Transplanted Neural Stem Cells Survive, Differentiate, and Improve Neurological Motor Function after Experimental Traumatic Brain Injury. Neurosurgery.

[22] Sinson G., Voddi M., McIntosh T.K. (1996). Combined fetal neural transplantation and nerve growth factor infusion: Effects on neurological outcome following fluid-percussion brain injury in the rat. J. Neurosurg..

[23] Park K.I., Teng Y.D., Snyder E.Y. (2002). The injured brain interacts reciprocally with neural stem cells supported by scaffolds to reconstitute lost tissue. Nat. Biotechnol..

[24] Shear D.A., Tate M.C., Archer D.R., Hoffman S.W., Hulce V.D., LaPlaca M.C., Stein D.G. (2004). Neural progenitor cell transplants promote long-term functional recovery after traumatic brain injury. Brain Res..

[25] Wennersten A., Meijer X., Holmin S., Wahlberg L., Mathiesen T. (2004). Proliferation, migration, and differentiation of human neural stem/progenitor cells after transplantation into a rat model of traumatic brain injury. J. Neurosurg..

[26] Harting M.T., Sloan L.E., Jimenez F., Baumgartner J., Cox C.S. (2009). Subacute Neural Stem Cell Therapy for Traumatic Brain Injury. J. Surg. Res..

[27] Kalladka D., Sinden J., Pollock K., Haig C., McLean J., Smith W., McConnachie A., Santosh C., Bath P.M., Dunn L. (2016). Human neural stem cells in patients with chronic ischaemic stroke (PISCES): A phase 1, first-in-man study. Lancet.

[28] Watanabe T.K. (2018). A Review of Stem Cell Therapy for Acquired Brain Injuries and Neurodegenerative Central Nervous System Diseases. PM&R.

[29] Krebsbach P.H., Kuznetsov S.A., Bianco P., Gehron Robey P. (1999). Bone Marrow Stromal Cells: Characterization and Clinical Application. Crit. Rev. Oral Biol. Med..

[30] Hass R., Kasper C., Böhm S., Jacobs R. (2011). Different populations and sources of human mesenchymal stem cells (MSC): A comparison of adult and neonatal tissue-derived MSC. Cell Commun. Signal..

[31] Li Y., Chen J., Chopp M. (2001). Adult Bone Marrow Transplantation after Stroke in Adult Rats. Cell Transplant..

[32] Chen J., Li Y., Wang L., Lu M., Zhang X., Chopp M. (2001). Therapeutic benefit of intracerebral transplantation of bone marrow stromal cells after cerebral ischemia in rats. J. Neurol. Sci..

[33] Mahmood A., Lu D., Li Y., Chen J.L., Chopp M. (2001). Intracranial bone marrow transplantation after traumatic brain injury improving functional outcome in adult rats. J. Neurosurg..

[34] Hu W., Jiang J., Yang F., Liu J. (2018). The impact of bone marrow-derived mesenchymal stem cells on neovascularisation in rats with brain injury. Folia Neuropathol..

[35] Guo S., Zhen Y., Wang A. (2017). Transplantation of bone mesenchymal stem cells promotes angiogenesis and improves neurological function after traumatic brain injury in mouse. Neuropsychiatr. Dis. Treat..

[36] Gao X., Deng P., Xu Z.C., Chen J. (2011). Moderate Traumatic Brain Injury Causes Acute Dendritic and Synaptic Degeneration in the Hippocampal Dentate Gyrus. PLoS ONE.

[37] Feng Y., Ju Y., Cui J., Wang L. (2017). Bone marrow stromal cells promote neuromotor functional recovery, via upregulation of neurotrophic factors and synapse proteins following traumatic brain injury in rats. Mol. Med. Rep..

[38] Mahmood A., Wang L., Chopp M. (2001). Treatment of Traumatic Brain Injury in Female Rats with Intravenous Administration of Bone Marrow Stromal Cells. Neurosurgery.

[39] Lu D., Li Y., Wang L., Chen J., Mahmood A., Chopp M. (2001). Intraarterial Administration of Marrow Stromal Cells in a Rat Model of Traumatic Brain Injury. J. Neurotrauma.

[40] Chopp M., Li Y. (2002). Treatment of neural injury with marrow stromal cells. Lancet Neurol..

[41] Lu D., Mahmood A., Wang L., Li Y., Lu M., Chopp M. (2001). Adult bone marrow stromal cells administered intravenously to rats after traumatic brain injury migrate into brain and improve neurological outcome. NeuroReport.

[42] Nichols J.E., Niles J.A., DeWitt D., Prough D., Parsley M., Vega S., Cantu A., Lee E., Cortiella J. (2013). Neurogenic and neuro-protective potential of a novel subpopulation of peripheral blood-derived CD133+ ABCG2+CXCR4+ mesenchymal stem cells: Development of autologous cell-based therapeutics for traumatic brain injury. Stem Cell Res. Ther..

[43] Lu D., Sanberg P.R., Mahmood A., Li Y., Wang L., Sanchez-Ramos J., Chopp M. (2002). Intravenous Administration of Human Umbilical Cord Blood Reduces Neurological Deficit in the Rat after Traumatic Brain Injury. Cell Transplant..

[44] Nagamura-Inoue T., He H. (2014). Umbilical cord-derived mesenchymal stem cells: Their advantages and potential clinical utility. World J. Stem Cells.

[45] Xiao J., Nan Z., Motooka Y., Low W.C. (2005). Transplantation of a Novel Cell Line Population of Umbilical Cord Blood Stem Cells Ameliorates Neurological Deficits Associated with Ischemic Brain Injury. Stem Cells Dev..

[46] Nan Z., Grande A., Sanberg C.D., Xiao F., Kuzmin-Nichols N., Low W.C., Cheeran M.C.J., Juliano M., Rotschafer J., Stone L.L.H. (2016). Amelioration of Ischemic Brain Injury in Rats with Human Umbilical Cord Blood Stem Cells: Mechanisms of Action. Cell Transplant..

[47] Zhang Z.X., Guan L.X., Zhang K., Zhang Q., Dai L.J. (2008). A combined procedure to deliver autologous mesenchymal stromal cells to patients with traumatic brain injury. Cytotherapy.

[48] Cox C.S., Baumgartner J.E., Harting M.T., Worth L.L., Walker P.A., Shah S.K., Ewing-Cobbs L., Hasan K.M., Day M.C., Lee D. (2011). Autologous Bone Marrow Mononuclear Cell Therapy for Severe Traumatic Brain Injury in Children. Neurosurgery.

[49] Cox C.S., Hetz R.A., Liao G.P., Aertker B.M., Ewing-Cobbs L., Juranek J., Savitz S.I., Jackson M.L., Romanowska-Pawliczek A.M., Triolo F. (2017). Treatment of Severe Adult Traumatic Brain Injury Using Bone Marrow Mononuclear Cells. Stem Cells.

[50] Tian C., Wang X., Wang X., Wang L., Wang X., Wu S., Wan Z. (2013). Autologous Bone Marrow Mesenchymal Stem Cell Therapy in the Subacute Stage of Traumatic Brain Injury by Lumbar Puncture. Exp. Clin. Transplant..

[51] Wang S., Cheng H., Dai G., Wang X., Hua R., Liu X., Wang P., Chen G., Yue W., An Y. (2013). Umbilical cord mesenchymal stem cell transplantation significantly improves neurological function in patients with sequelae of traumatic brain injury. Brain Res..

[52] Laskowitz D.T., Bennett E.R., Durham R.J., Volpi J.J., Wiese J.R., Frankel M., Shpall E., Wilson J.M., Troy J., Kurtzberg J. (2018). Allogeneic Umbilical Cord Blood Infusion for Adults with Ischemic Stroke: Clinical Outcomes from a Phase I Safety Study. Stem Cells Transl. Med..

[53] Kelso M.L., Gendelman H.E. (2014). Bridge between neuroimmunity and traumatic brain injury. Curr. Pharm. Des..

[54] Krämer T.J., Hack N., Brühl T.J., Menzel L., Hummel R., Griemert E.V., Klein M., Thal S.C., Bopp T., Schäfer M.K.E. (2019). Depletion of regulatory T cells increases T cell brain infiltration, reactive astrogliosis, and interferon-γ gene expression in acute experimental traumatic brain injury. J. Neuroinflamm..

[55] Bjarnadóttir K., Benkhoucha M., Merkler D., Weber M.S., Payne N.L., Bernard C.C.A., Molnarfi N., Lalive P.H. (2016). B cell-derived transforming growth factor-β1 expression limits the induction phase of autoimmune neuroinflammation. Sci. Rep..

[56] Sîrbulescu R.F., Boehm C.K., Soon E., Wilks M.Q., Ilieş I., Yuan H., Maxner B., Chronos N., Kaittanis C., Normandin M.D. (2017). Mature B cells accelerate wound healing after acute and chronic diabetic skin lesions. Wound Repair Regen..

[57] Sîrbulescu R.F., Chung J.Y., Edmiston W.J., Poznansky S.A., Poznansky M.C., Whalen M.J. (2019). Intraparenchymal Application of Mature B Lymphocytes Improves Structural and Functional Outcome after Contusion Traumatic Brain Injury. J. Neurotrauma.

[58] Hu J., Chen L., Huang X., Wu K., Ding S., Wang W., Wang B., Smith C., Ren C., Ni H. (2019). Calpain inhibitor MDL28170 improves the transplantation-mediated therapeutic effect of bone marrow-derived mesenchymal stem cells following traumatic brain injury. Stem Cell Res. Ther..

[59] Bendall L.J., Bradstock K.F. (2014). G-CSF: From granulopoietic stimulant to bone marrow stem cell mobilizing agent. Cytokine Growth Factor Rev..

[60] Acosta S.A., Tajiri N., Shinozuka K., Ishikawa H., Sanberg P.R., Sanchez-Ramos J., Song S., Kaneko Y., Borlongan C.V. (2014). Combination therapy of human umbilical cord blood cells and granulocyte colony stimulating factor reduces histopathological and motor impairments in an experimental model of chronic traumatic brain injury. PLoS ONE.

[61] Devetzi M., Goulielmaki M., Khoury N., Spandidos D.A., Sotiropoulou G., Christodoulou I., Zoumpourlis V. (2018). Genetically-modified stem cells in treatment of human diseases: Tissue kallikrein (KLK1)-based targeted therapy (Review). Int. J. Mol. Med..

[62] Peruzzaro S.T., Andrews M.M.M., Al-Gharaibeh A., Pupiec O., Resk M., Story D., Maiti P., Rossignol J., Dunbar G.L. (2019). Transplantation of mesenchymal stem cells genetically engineered to overexpress interleukin-10 promotes alternative inflammatory response in rat model of traumatic brain injury. J. Neuroinflamm..

[63] van Velthoven C.T., Braccioli L., Willemen H.L., Kavelaars A., Heijnen C.J. (2014). Therapeutic potential of genetically modified mesenchymal stem cells after neonatal hypoxic-ischemic brain damage. Mol. Ther. J. Am. Soc. Gene Ther..

